# Assessment of automated analysis of portable oximetry as a screening test for moderate-to-severe sleep apnea in patients with chronic obstructive pulmonary disease

**DOI:** 10.1371/journal.pone.0188094

**Published:** 2017-11-27

**Authors:** Ana M. Andrés-Blanco, Daniel Álvarez, Andrea Crespo, C. Ainhoa Arroyo, Ana Cerezo-Hernández, Gonzalo C. Gutiérrez-Tobal, Roberto Hornero, Félix del Campo

**Affiliations:** 1 Pneumology Service, Río Hortega University Hospital, Valladolid, Spain; 2 Biomedical Engineering Group, University of Valladolid, Valladolid, Spain; Istituti Clinici Scientifici Maugeri, ITALY

## Abstract

**Background:**

The coexistence of obstructive sleep apnea syndrome (OSAS) and chronic obstructive pulmonary disease (COPD) leads to increased morbidity and mortality. The development of home-based screening tests is essential to expedite diagnosis. Nevertheless, there is still very limited evidence on the effectiveness of portable monitoring to diagnose OSAS in patients with pulmonary comorbidities.

**Objective:**

To assess the influence of suffering from COPD in the performance of an oximetry-based screening test for moderate-to-severe OSAS, both in the hospital and at home.

**Methods:**

A total of 407 patients showing moderate-to-high clinical suspicion of OSAS were involved in the study. All subjects underwent (i) supervised portable oximetry simultaneously to in-hospital polysomnography (PSG) and (ii) unsupervised portable oximetry at home. A regression-based multilayer perceptron (MLP) artificial neural network (ANN) was trained to estimate the apnea-hypopnea index (AHI) from portable oximetry recordings. Two independent validation datasets were analyzed: COPD *versus* non-COPD.

**Results:**

The portable oximetry-based MLP ANN reached similar intra-class correlation coefficient (ICC) values between the estimated AHI and the actual AHI for the non-COPD and the COPD groups either in the hospital (non-COPD: 0.937, 0.909–0.956 CI95%; COPD: 0.936, 0.899–0.960 CI95%) and at home (non-COPD: 0.731, 0.631–0.808 CI95%; COPD: 0.788, 0.678–0.864 CI95%). Regarding the area under the receiver operating characteristics curve (AUC), no statistically significant differences (*p* >0.01) between COPD and non-COPD groups were found in both settings, particularly for severe OSAS (AHI ≥30 events/h): 0.97 (0.92–0.99 CI95%) non-COPD vs. 0.98 (0.92–1.0 CI95%) COPD in the hospital, and 0.87 (0.79–0.92 CI95%) non-COPD vs. 0.86 (0.75–0.93 CI95%) COPD at home.

**Conclusion:**

The agreement and the diagnostic performance of the estimated AHI from automated analysis of portable oximetry were similar regardless of the presence of COPD both in-lab and at-home. Particularly, portable oximetry could be used as an abbreviated screening test for moderate-to-severe OSAS in patients with COPD.

## Introduction

Obstructive sleep apnea syndrome (OSAS) patients suffer from recurrent episodes of airflow limitation due to intermittent complete or partial collapse of the upper airway while sleeping, leading to non-restful sleep and diminished quality of life [1, 2]. The burden of OSAS is continuously increasing mainly due to the drawbacks of the standard diagnostic methodology, i.e. in-hospital polysomnography (PSG). While being effective, the availability and accessibility of PSG is very limited and it is considered labor-intensive and expensive [3–5]. In order to overcome these limitations, several abbreviated tests for OSAS detection have been proposed during the last years. The use of portable monitors at home focused the attention of many researchers due to their readiness, simplicity, efficiency, and lower cost [4, 6–10]. Nevertheless, most researchers exclude patients with significant cardiovascular and pulmonary comorbidities from the population under study, which is an important limitation in order to generalize the results. In fact, a recent report of the American Academy of Sleep Medicine (AASM) does not recommend the use of portable monitoring for OSAS screening in such patients because there is still little if any evidence on its effectiveness [11]. However, it is known that the prevalence of both cardiovascular and pulmonary comorbidities is high among sleep apnea patients [12, 13]. Moreover, sleep apnea has been closely related with significant decreased health status and quality of life in the presence of such conditions [12–15]. Therefore, there is currently an increasing demand for studies focusing on the assessment of home testing algorithms in patients showing significant comorbidities, especially chronic obstructive pulmonary disease (COPD) [10, 16, 17].

The coexistence of OSAS and COPD, the so-called overlap syndrome, leads to major social and healthcare-related consequences, mostly in the context of cardiovascular disease [15]. Patients showing both conditions simultaneously suffer from more severe oxygen desaturations during sleep than those with either COPD or OSAS alone, as well as worse daytime hypoxemia and hypercapnia [13, 15]. Therefore, an early diagnosis of OSAS is essential in order to receive an effective treatment and reduce mortality. The revised Global Initiative for Chronic Obstructive Lung Disease (GOLD) guidelines highlight the need for controlling the impact of comorbid conditions [18]. Particularly, screening for sleep-related breathing disorders is strongly recommended in COPD patients showing common daytime and/or night symptoms, such as hypersomnolence and frequent sleep arousals [13, 15, 19].

There are very few studies focused on the evaluation of portable oximetry as an abbreviated test for OSAS detection among patients with concomitant COPD [20–22]. These studies analyzed small datasets using substantially different methodologies and settings. As a result, contradictory findings are reported. Therefore, further research is needed.

Artificial neural networks (ANNs) have demonstrated to be very useful in many applications of medicine and, particularly, in the field of OSAS diagnosis [23–27]. In a previous study by our group, we assessed the diagnostic performance of an ANN trained to estimate the apnea-hypopnea index (AHI) of patients suspected of suffering from OSAS using the oximetry signal from a controlled PSG carried out in the hospital [26]. In the present study, we propose to use an ANN to estimate the AHI from portable nocturnal oximetry in the presence of COPD. Accordingly, our main goal was to design and exhaustively validate the effectiveness of an ANN-based automated test, assessing how the presence of COPD influences the diagnostic performance of portable oximetry monitoring.

## Materials and methods

### Population under study

**Fig 1:**
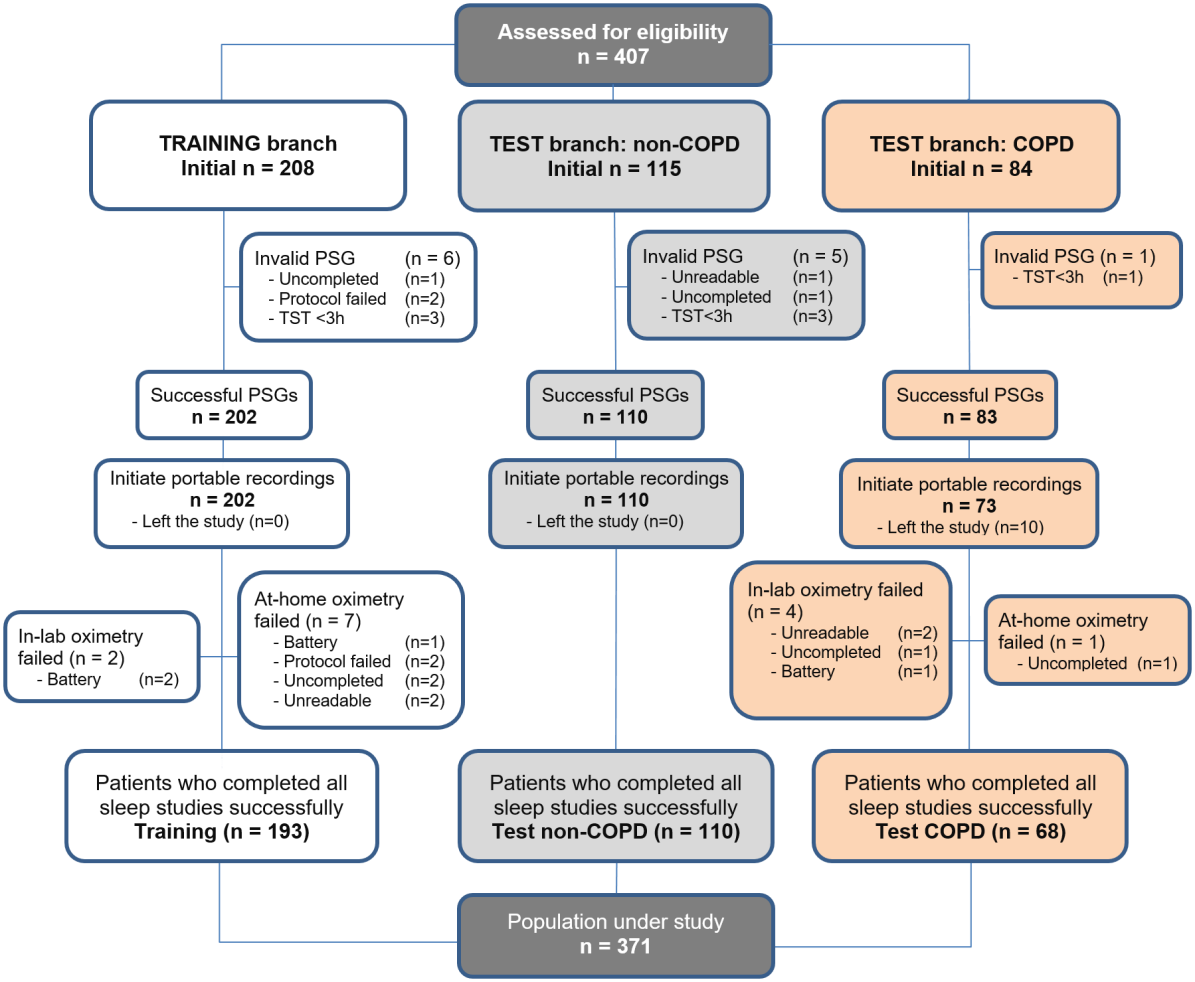
Patient recruitment flowchart. PSG: polysomnography; TST: total sleep time.

Fig 1 shows the detailed flowchart of the study. Three patient groups were recruited from June 2013 to January 2015 in order to develop our research. Firstly, consecutive patients referred to the sleep unit and regardless of suffering from COPD composed our initial training dataset. Secondly, consecutive patients without COPD referred to the sleep unit composed the non-COPD validation dataset. Finally, consecutive patients referred to the Pneumology outpatient facilities due to COPD and also showing clinical suspicion of OSAS composed the COPD validation dataset. Regarding COPD patients, subjects aged ≥35 years old, current or ex-smokers with a smoking history of at least 10 pack/years were considered. At the time of COPD diagnosis, complete pulmonary function evaluation (Master screen PFT, Jaeger) was conducted to confirm the disease, including pre- and post-bronchodilator spirometry, lung volumes, and lung diffusion capacity. According to GOLD [18], patients showing a post-bronchodilator spirometry with forced expired volume in 1 second to forced vital capacity ratio (FEV_1_/FVC) <70% were involved in the study. Patients with a previous diagnosis and/or treatment for OSAS, additional sleep disorders, and severe cardiovascular diseases were excluded.

The Ethics Committee of the Río Hortega University Hospital approved the study (approval number: CEIC 7/13), which was conducted according to the principles expressed in the Declaration of Helsinki. All patients were informed to participate in the study and signed an informed consent.

### Data collection protocol and sleep studies

All subjects underwent two sleep studies during consecutive nights: (i) supervised portable oximetry simultaneously to in-hospital PSG and (ii) unsupervised portable oximetry at home. The sequence of the attended/unattended sleep studies was determined randomly. The AHI from in-lab PSG (AHI_PSG_) was used to confirm OSAS. In addition, two different estimations of the AHI were computed from portable oximetry using the proposed ANN: (i) estimated AHI from supervised oximetry in the sleep laboratory (AHI_OX-LAB_) and (ii) estimated AHI from unattended oximetry at home (AHI_OX-HOME_).

Standard in-lab PSG was carried out using a polysomnograph E-series by Compumedics (Compumedics Limited, Victoria, Australia). Electroencephalogram (F3-A2, F4-A1, C3-A2, C4-A1, Cz-A2, O1-A2, O2-A1), electrooculogram (right and left), electromyogram (chin and both tibias), electrocardiogram, respiratory effort (chest and abdominal), respiration (thermistor and nasal pressure), oximetry (SpO_2_ and pulse rate), body position, snoring sounds, and video were recorded and stored for offline inspection by a single trained specialist. The AASM rules were used to score sleep studies and derive the AHI from each PSG [28]. All PSGs with a total sleep time (TST) <3 h due to significant signal loss (artifacts), voluntary termination of the study by the patient, or insufficient data to assess sleep (low sleep efficiency and/or no REM sleep), were withdrawn from the study [29].

The Nonin WristOx2 3150 (Nonin Medical, Inc., Plymouth, MI, USA) was used to perform portable oximetry both in the hospital facilities and at patients’ home. The SpO_2_ signal were recorded using a finger probe at a sampling rate of 1 sample per second (1 Hz). Both oximetry probes, the one corresponding to the PSG and that corresponding to the portable device, were placed on consecutive fingers of the same hand. Previously to the unsupervised recording at home, all participants received both verbal and written instructions on how to use the oximeter. All portable SpO_2_ recordings with a total recording time (TRT) <4 h due to significant signal loss (artifacts), premature battery depletion, or voluntary termination of the recording by the patient, were discarded [30]. Additionally, patients who did not complete both in-hospital and at-home studies were also withdrawn from the study.

### Automated analysis of SpO_2_ from oximetry

#### Pre-processing

SpO_2_ recordings were downloaded from the portable oximeter and processed offline. Each recording was scanned and all zero samples and transient deeps due to patient’s movements were removed. Additionally, all changes between consecutive samples greater than or equal to 4%/s as well as saturation samples below 20% were considered artifacts and removed from the signal [31]. When large segments of consecutive artifacts were removed, the remaining valid sections were linked. Linear interpolation was applied if the difference between concatenated samples was ≥4%/s in order to avoid the inclusion of non-natural changes in the SpO_2_ time series.

#### Feature extraction

Every overnight oximetric profile was characterized using an initial set of features from complementary approaches (time *vs*. frequency and linear *vs*. nonlinear), which has demonstrated to be very useful in the context of automated OSAS diagnosis [26, 32–34]. A total of 16 variables arranged into 4 feature subsets were computed: time-domain statistics, frequency-domain statistics, conventional spectral measures, and nonlinear measures. Statistics and nonlinear methods in the time domain were applied to non-overlapping 512-sample length segments. Statistics and conventional measures in the frequency domain were derived from the power spectral density (PSD) function. The widely applied Welch’s method (512-sample Hanning window, 50% overlapping, 1024-sample fast Fourier transform) were used to estimate the spectral content of each recording [35].

**Time-domain statistics.** The position (central tendency) and shape (width, asymmetry, and peakedness) of the normalized data histogram of saturation amplitudes from each oximetric time series were parameterized by means of the 1^st^- to 4^th^-order statistical moments [36], i.e., mean (*M1t*), variance (*M2t*), skewness (*M3t*), and kurtosis (*M4t*).

**Frequency-domain statistics.** Similarly, the data histogram of amplitudes from each normalized PSD function was characterized using the 1^st^- to 4^th^-order statistical moments, i.e., mean (*M1f*), variance (*M2f*), skewness (*M3f*), and kurtosis (*M4f*) in the frequency domain. In addition, the median frequency (*MF*) and the spectral entropy (*SE*) [37] of each PSD were also computed to further characterize the power distribution of the spectrum.

**Conventional spectral measures.** Common amplitude- and power-based measures were also computed to characterize the severity and recurrence of desaturations. The total signal power (*P*_*T*_) as well as the peak amplitude (*PA*) and relative power (*P*_*R*_) in the frequency band of interest for adult sleep apnea (0.014–0.033 Hz) were obtained [29].

**Nonlinear measures.** Sample entropy (*SampEn*) [38], central tendency measure (*CTM*) [39], and Lempel-Ziv complexity (*LZC*) [40] were applied to quantify the irregularity, variability, and complexity of each SpO_2_ recording, respectively.

#### Feature selection

The 16 features derived from every oximetric recording compose a feature pattern that characterizes the presence of OSAS on each subject. As mentioned, this initial feature set comprises valuable information linked with the disease. Nevertheless, an improved as well as reduced feature subset can be derived by applying a feature selection algorithm. Previous works have shown that dimensionality reduction algorithms enhances the prediction ability of oximetric features in the context of OSAS diagnosis [32–34].

In this study, the fast correlation-based filter (FCBF), a filter methodology for feature selection independent of the pattern recognition technique, is applied [41]. FCBF has been previously assessed in the context of automated analysis of supervised airflow recordings for OSAS detection [42, 43]. FCBF identifies automatically the most relevant and non-redundant features in terms of the symmetrical uncertainty (SU) [41]. SU is a normalization of the so-called information gain (IG), which is a measure of predictability based on the information shared between variables. Two main variable filtering stages are involved: (i) relevance- and (ii) redundancy-based feature selection. Regarding the relevance analysis, the association between each input feature and the severity of the disease was estimated. To achieve this goal, the SU between each feature (*X*_*i*_) and the standard AHI from PSG (*Y*) was computed as follows [41]:

$$SU_{i}(X_{i},Y)=2\frac{IG_{i}(X_{i},Y)}{H(X_{i})+H(Y)},\;i=1,\ldots ,\;p,$$ (1)

where *H* refers to the widely known Shannon’s entropy. According to their degree of relevance, original input features are ranked from the most (higher *SU*_*i*_) to the less (lower *SU*_*i*_) relevant. Then, in order to implement the redundancy analysis, *SU*_*i*,*j*_ is computed between each pair of ranked features starting from the most relevant one (*i*, *j* = 1, …, *p*; *i* > *j* in the ranking). When *SU*_*i*,*j*_ ≥ *SU*_*i*_, the feature *j* is removed due to redundancy. Hence, the final optimum feature subset was composed of the most relevant and non-redundant variables from portable oximetry.

#### Pattern recognition

ANNs are composed of multiple interconnected nodes, the so-called neurons, arranged in consecutive levels (layers) leading to a highly parallel structure [44]. In the OSAS framework, a regression-based ANN is able to estimate the AHI, which is a continuous scalar positive magnitude. Accordingly, a single output neuron is used to approximate the target AHI and a linear activation function ranging [0, ∞) is applied to model the regression problem. Therefore, the output of a regression MLP ANN is given by [45]:

$$y=f(\text{x},\text{w})=\sum_{j=1}^{N_{H}}\left[\omega_{j}g_{t}\left(\sum_{i=1}^{d}\omega_{ij}x_{i}+b_{j}\right)+b\right],$$ (2)

where *d* is the number of features in the optimum input pattern from the previous feature selection process, *N*_*H*_ is the number of neurons in the hidden layer, ω_*j*_ is the weight connecting the hidden neuron *h*_*j*_ and the output, *b* is a bias term linked with the output of the network, ω_*ij*_ is the weight connecting the feature *i* from the input pattern vector with the hidden neuron *h*_*j*_, *b*_*j*_ is the bias of the neuron *h*_*j*_ in the hidden layer, and *g*_*t*_(·) is the activation function of the neurons in the hidden layer. Weights and biases are determined automatically during the iterative learning process according to the maximum likelihood principle [44]. In order to avoid overfitting, the widely known weight decay regularization technique was applied [44].

Model selection, i.e., optimization of the user-dependent input parameters (number of hidden neurons and regularization), was accomplished by leave-one-out cross-validation (loo-cv) in the training dataset. The loo-cv procedure was repeated 100 times to avoid a potential bias linked with the random initialization of weights. The average intra-class correlation coefficient (ICC) between the estimated AHI and the actual AHI from PSG was used as the metric for model selection. Next, the whole training set was used to carry out the learning process. Finally, the trained MLP ANN was further assessed using two independent test datasets composed of unseen patients: the non-COPD group and the COPD group.

### Statistical analysis

The software tools Matlab version R2014a and IBM SPSS version 19 were used for performing signal processing and statistical analyses, respectively. A descriptive analysis of clinical and polysomnographic variables of the population under study was accomplished by computing the median and interquartile range (IQR). The non-parametric Kruskal-Wallis test was used to search for significant differences among the 3 groups under study for continuous variables, whereas the Chi^2^ test was used for the categorical ones. A *p*-value <0.01 was considered significant. The ICC was computed to measure quantitatively the agreement between the estimated AHI and the actual AHI from PSG. In addition, Bland-Altman and Mountain plots were used to assess qualitatively the agreement between both indices taking into account the groups (COPD and non-COPD) and the settings (in-hospital and at-home) under study.

Regarding diagnostic performance assessment, common cutoffs for moderate (AHI ≥15 events/h) and severe (AHI ≥30 events/h) OSAS were studied. The following widely known metrics were computed for each cutoff in the independent test datasets: sensitivity (Se), specificity (Sp), positive predictive value (PPV), negative predictive value (NPV), positive likelihood ratio (LR+), negative likelihood ratio (LR-), and accuracy (Acc). Additionally, the area under the receiving operating characteristics curve (AUC) was computed as overall performance measure independent of a particular cutoff. The 95% confidence interval (CI95%) was computed for every metric. The recommendations of the STARD task force for reporting diagnostic performance measures in medical research were taken into account [46]. Conventional oxygen desaturation indices (ODIs) of 3% (ODI3) and 4% (ODI4) were assessed for comparison purposes.

## Results

A total of 407 eligible patients were involved in the study. Fig 1 shows a detailed description of the recruitment process. Regarding patients withdrawn from the study, 12 PSGs (2.95%) were discarded due to insufficient total sleep time (TST <3 hours), uncompleted PSG (the patient left the study or did not attend to the sleep unit), or because files were corrupted. Similarly, 14 portable SpO_2_ recordings (3.44%) were removed due to reasons (either technical or human) linked with portable oximetry: 6 supervised in-hospital oximetry (1.45%) and 8 unsupervised at home (1.97%). Finally, 193 patients with suspicion of suffering from OSAS and regardless of COPD composed the training dataset, whereas 110 patients composed the non-COPD test set and 68 patients composed the COPD test group.

Anthropometric and clinical characteristics of the population under study are summarized in Table 1. Table 2 shows the polysomnographic variables of every group under study. Significant statistical differences between groups were found for CT90, basal, minimum, and average saturation, whereas no significant differences were identified for the ODI3. Regarding sleep staging and respiratory-related events, no significant statistical differences were found among patient groups.

**Table 1:** Demographic and clinical characteristics of the patient groups under study.

Feature	Training	Test 1: non-COPD	Test 2: COPD	*p*-value
Subjects (n)	193	110	68	-
Age (years)	55.0 [18.2]	55.0 [18.0]	64.5 [11.0]	<0.01
Males (n)	148 (76.7%)	76 (69.1%)	60 (88.2%)	<0.01
BMI (Kg/m^2^)	28.3 [6.2]	28.6 [5.8]	29.0 [5.4]	0.739
AHI (events/h)	33.6 [43.8]	33.8 [41.1]	37.8 [45.1]	0.609
AHI ≥5 events/h (n)	174 (90.2%)	101 (91.8%)	67 (98.5%)	0.085
AHI ≥15 events/h (n)	143 (74.1%)	81 (73.6%)	52 (76.5%)	0.907
AHI ≥30 events/h (n)	108 (56.0%)	63 (57.3%)	39 (57.4%)	0.966

Data are presented as median [interquartile range] for quantitative variables whereas n(%) is used for categorical variables. Non-COPD: test dataset composed of patients without chronic obstructive pulmonary disease; COPD: test dataset composed of chronic obstructive pulmonary disease patients; BMI: body mass index; AHI: apnea-hypopnea index.

**Table 2:** Polysomnographic variables of the groups under study: Sleep staging, respiratory related events, and oximetric indexes.

Feature	Training	Test 1: non-COPD	Test 2: COPD	*p*-value
Sleep efficiency (%)	83.7 [16.6]	84.9 [16.9]	77.1 [23.5]	0.011
Stage N1 (%)	14.4 [9.7]	12.8 [15.2]	16.2 [14.2]	0.076
Stage N2 (%)	32.1 [13.4]	32.4 [14.5]	28.6 [12.4]	0.053
Stage N3 (%)	35.8 [17.6]	35.2 [21.7]	39.0 [17.8]	0.337
REM sleep (%)	15.4 [9.6]	14.4 [8.9]	14.6 [9.3]	0.665
Arousal index (events/h)	30.5 [22.0]	25.8 [22.9]	30.7 [15.6]	0.061
AHI (events/h)	33.6 [43.8]	33.8 [41.1]	37.8 [45.1]	0.609
AI (events/h)	10.9 [30.8]	10.6 [27.9]	9.0 [28.1]	0.934
ODI3 (events/h)	32.7 [41.3]	35.5 [38.2]	38.3 [43.1]	0.509
CT90 (%)	10.5 [25.2]	10.1 [29.9]	46.2 [66.0]	<<0.01
SpO_2_ basal	94.0 [2.0]	94.0 [2.0]	91.0 [3.0]	<<0.01
SpO_2_ min	82.0 [13.0]	81.0 [10.0]	78.0 [11.0]	<0.01
SpO_2_ mean	93.0 [3.0]	93.0 [3.0]	90.0 [4.0]	<<0.01

Data are presented as median [interquartile range]. Non-COPD: test dataset composed of patients without chronic obstructive pulmonary disease; COPD: test dataset composed of chronic obstructive pulmonary disease patients; REM: rapid eye movement sleep; AHI: apnea-hypopnea index; ODI3: oxygen desaturation index of 3% in the PSG; CT90: percentage of the total sleep time in the PSG with a saturation below 90%; SpO_2_: blood oxygen saturation; min: minimum value in the overall recording.

Table 3 summarizes the characteristics from spirometry of the COPD group. According to GOLD stages [18], 23.5% were categorized as GOLD1, 57.4% as GOLD2, 17.6% as GOLD3, and 1.5% as GOLD4.

**Table 3:** Common pulmonary functional measures of COPD patients derived from post-bronchodilator spirometry.

Measure from post-bronchodilator spirometry	Median [IQR]
FVC (L)	2.9 [1.4]
FVC (%)	81.8 [27.4]
FEV_1_ (liters)	1.7 [0.9]
FEV_1_ (%)	63.7 [24.4]
FEV_1_/FVC	60.6 [14.6]
FVC improvement	4.0 [7.9]
FEV_1_ improvement	4.0 [8.3]

Data are presented as median [interquartile range]. IQR: interquartile range; FEV_1_: forced expiratory volume in one second; FVC: forced vital capacity.

### ANN design in the training dataset

#### In-hospital supervised setting

**Fig 2:**
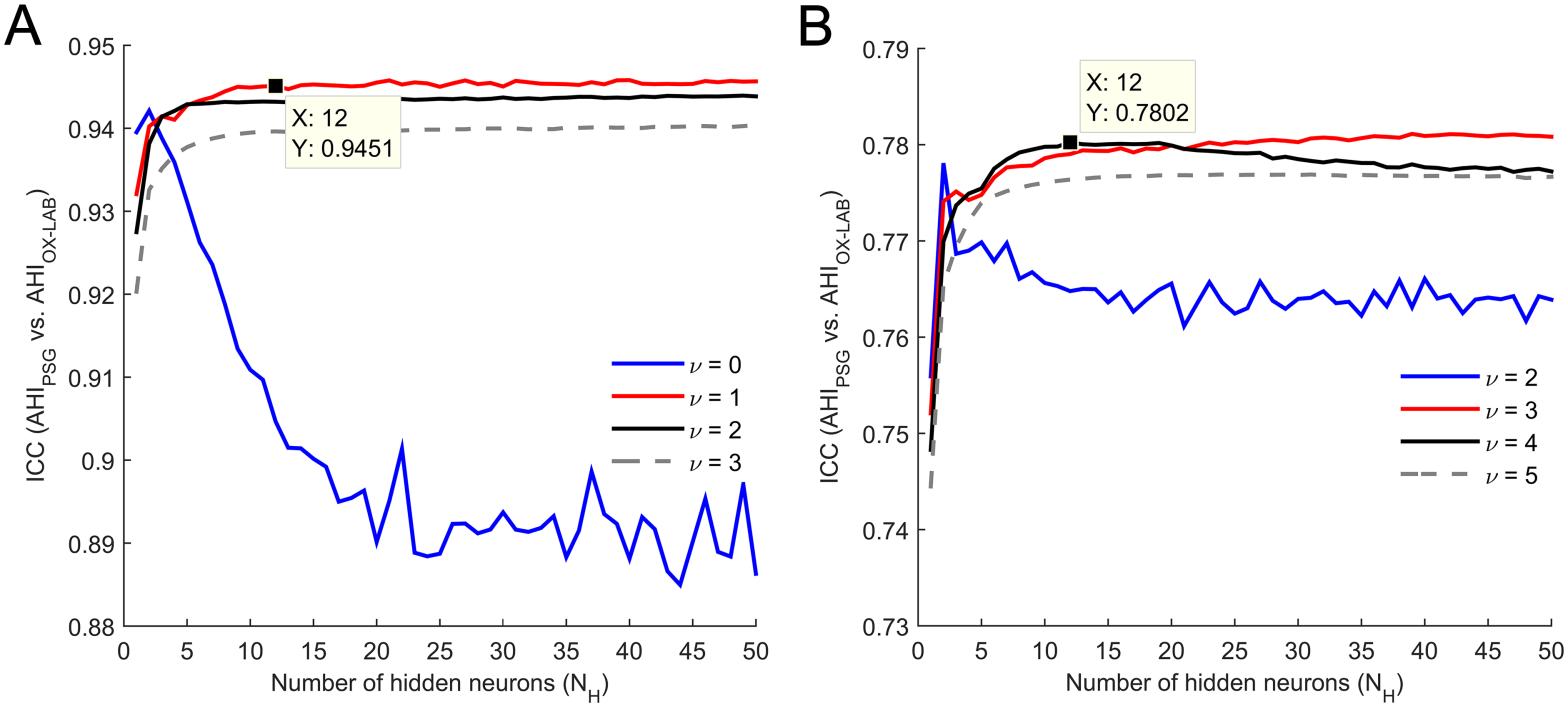
Optimization (model selection) of the MLP ANN in the training set. (A) In-hospital supervised monitoring. (B) At-home unattended monitoring. ICC: intra-class correlation coefficient; N_H_: number of neurons in the hidden layer; *ν*: regularization parameter.

A total of 9 features were automatically selected from in-lab attended recordings: *M1t*, *M2t*, *M3t*, *M4t*, *MF*, *P*_*R*_, *SampEn*, *CTM*, and *LZC*. Fig 2A shows the model selection process for the ANN in the training set using these features. The number of neurons in the hidden layer of the optimum ANN from supervised oximetry was *N*_*H*_ = 12 and the regularization parameter was set to *ν* = 1. The proposed ANN achieved 0.96 ICC (0.95, 0.97 CI95%) in the whole training dataset.

#### At-home unsupervised setting

Similarly, a total of 8 features composed the optimum feature subset from unattended recordings at home: *M1t*, *M3t*, *M4t*, *SE*, *P*_*R*_, *SampEn*, *CTM*, and *LZC*. Fig 2B shows the model selection procedure. The optimum ANN from unsupervised oximetry contained *N*_*H*_ = 12 hidden neurons and the regularization parameters was *ν* = 4. The proposed ANN reached 0.80 ICC (0.75, 0.85 CI95%) in the whole training set.

### Performance assessment in the test datasets

#### Agreement with AHI from PSG

Table 4 shows the ICC values achieved by the proposed oximetry-based ANNs and conventional ODI3 and ODI4. Regarding the AHI_OX-LAB_ from portable oximetry in the hospital setting, ICC values were very similar in the non-COPD (0.937, CI95% 0.909–0.956) and in the COPD (0.936, CI95% 0.899–0.960) groups (S1 and S2 Tables). In the same way, ICC values corresponding to AHI_OX-HOME_ from at-home recordings were again similar among non-COPD (0.731, CI95% 0.631–0.808) and COPD (0.788, CI95% 0.678–0.864) patients (S1 and S2 Tables). It is important to note that the estimated AHI_OX-LAB_ and AHI_OX-HOME_ reached notably higher agreement with the actual AHI from PSG than conventional ODIs in both settings.

**Table 4:** Agreement between estimated AHI using the proposed ANN and conventional ODIs with actual AHI from PSG for the groups and settings under study.

Setting	In-laboratory		At-home	
Group	non-COPD	COPD	non-COPD	COPD
Estimated AHI	0.937	0.936	0.731	0.788
	(0.909, 0.956)	(0.899, 0.960)	(0.631, 0.808)	(0.678, 0.864)
ODI3	0.904	0.892	0.536	0.670
	(0.634, 0.960)	(0.664, 0.952)	(0.100, 0.751)	(0.126, 0.856)
ODI4	0.745	0.693	0.403	0.518
	(0, 0.911)	(0, 0.819)	(0, 0.676)	(0, 0.788)

Data are presented as magnitude and 95% confidence interval. non-COPD: test dataset composed of patients without chronic obstructive pulmonary disease; COPD: test dataset composed of chronic obstructive pulmonary disease patients; AHI: apnea-hypopnea index; ODI3: oxygen desaturation index of 3% from portable oximetry; ODI4: oxygen desaturation index of 4% from portable oximetry.

**Fig 3:**
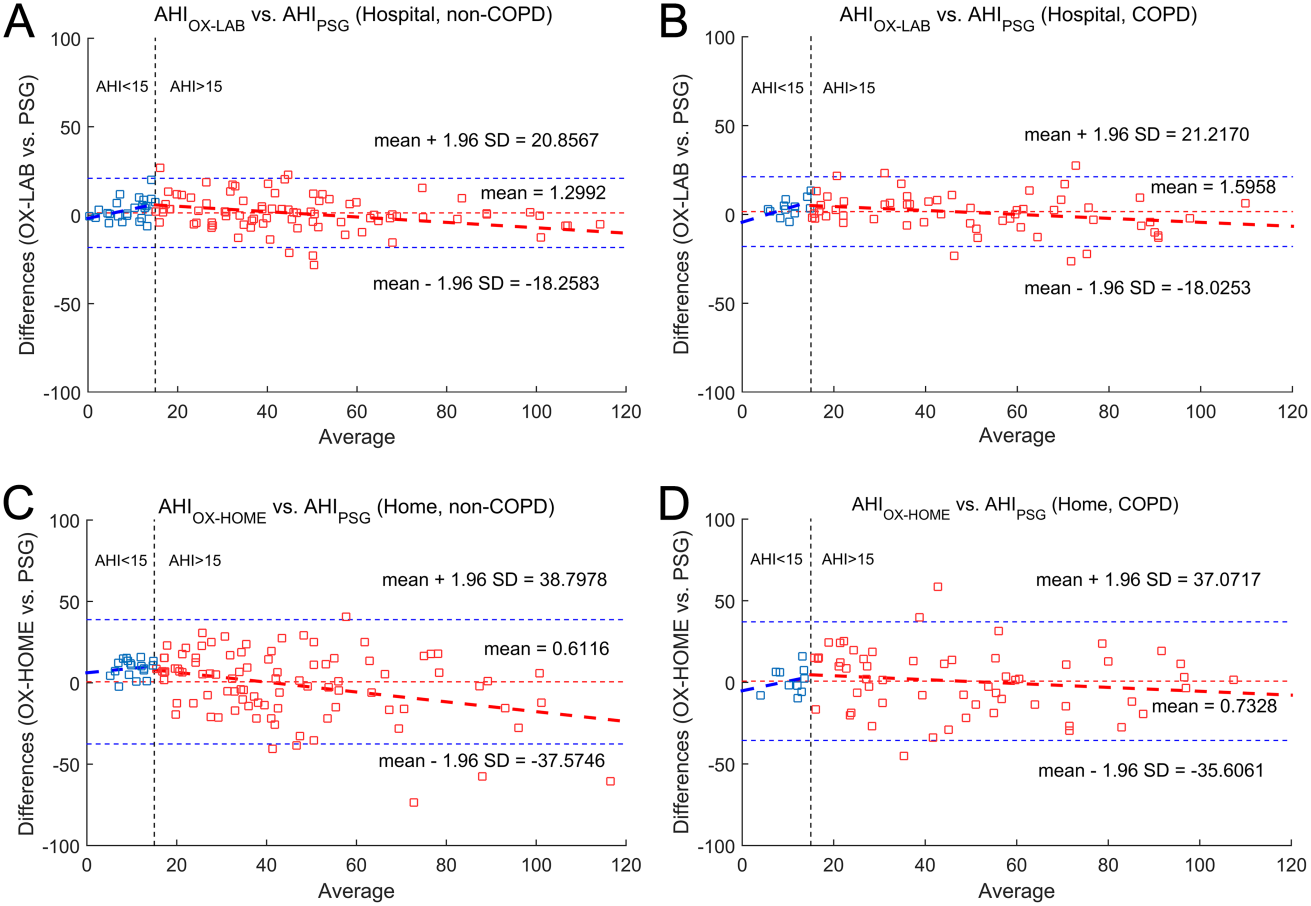
Bland-Altman plots showing agreement between estimated AHI from nocturnal oximetry and actual AHI from PSG. (A) Supervised oximetry in the laboratory for non-COPD subjects. (B) Supervised oximetry in the laboratory for COPD patients. (C) Unattended oximetry at home for non-COPD subjects. (D) Unattended oximetry at home for COPD patients. AHI_OX-LAB_: apnea-hypopnea index from in-hospital oximetry; PSG: polysomnography; in-LAB: supervised setting in the hospital; non-COPD: patients without chronic obstructive pulmonary disease; COPD: patients with chronic obstructive pulmonary disease; AHI_OX-HOME_: apnea-hypopnea index from at-home oximetry; at-HOME: supervised setting at home.

**Fig 4:**
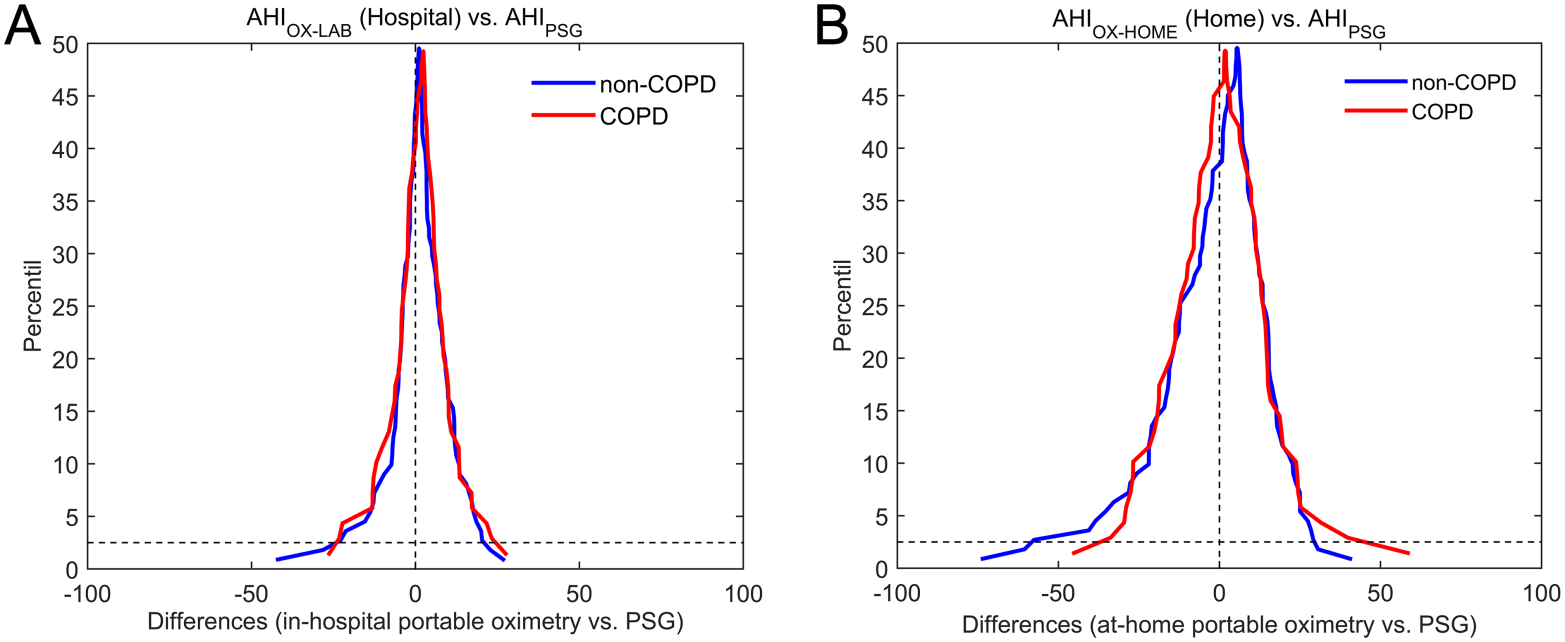
Mountain plots showing differences between the reference AHI from PSG and the estimated AHI of non-COPD and COPD groups. (A) Supervised portable oximetry in the hospital simultaneous to PSG. (B) Unattended portable oximetry at home in a different night. AHI_OX-LAB_: apnea-hypopnea index from in-hospital oximetry; PSG: polysomnography; non-COPD: patients without chronic obstructive pulmonary disease; COPD: patients with chronic obstructive pulmonary disease; AHI_OX-HOME_: apnea-hypopnea index from at-home oximetry.

Fig 3A–3D depict the Bland-Altman agreement plots for the estimated AHI and the standard AHI for the groups under study. In the hospital, we can observe in Fig 3A and 3B that the AHI_OX-LAB_ presented a symmetrical behavior and similar dispersion (limits of agreement) in the non-COPD group (confidence interval = 39.12) and in the COPD dataset (confidence interval = 39.24). In addition, the AHI_OX-LAB_ showed low bias, i.e., mean difference with the actual AHI, in both groups (non-COPD: 1.30; COPD: 1.60). Regarding the data derived from unsupervised recordings at home, the Bland-Altman agreement plots in Fig 3C and 3D show similar dispersion in both datasets (confidence interval: 76.37 in non-COPD and 72.68 in COPD) as well as comparable low bias (averaged mean differences: 0.61 in non-COPD and 0.73 in COPD) for the AHI_OX-HOME_. It is important to highlight that no significant differences were found between non-COPD and COPD groups neither in the hospital (*p* = 0.33) nor at home (*p* = 0.48). Mountain plots depicted in Fig 4A and 4B confirm these findings.

#### Diagnostic performance as a screening test for OSAS

**Fig 5:**
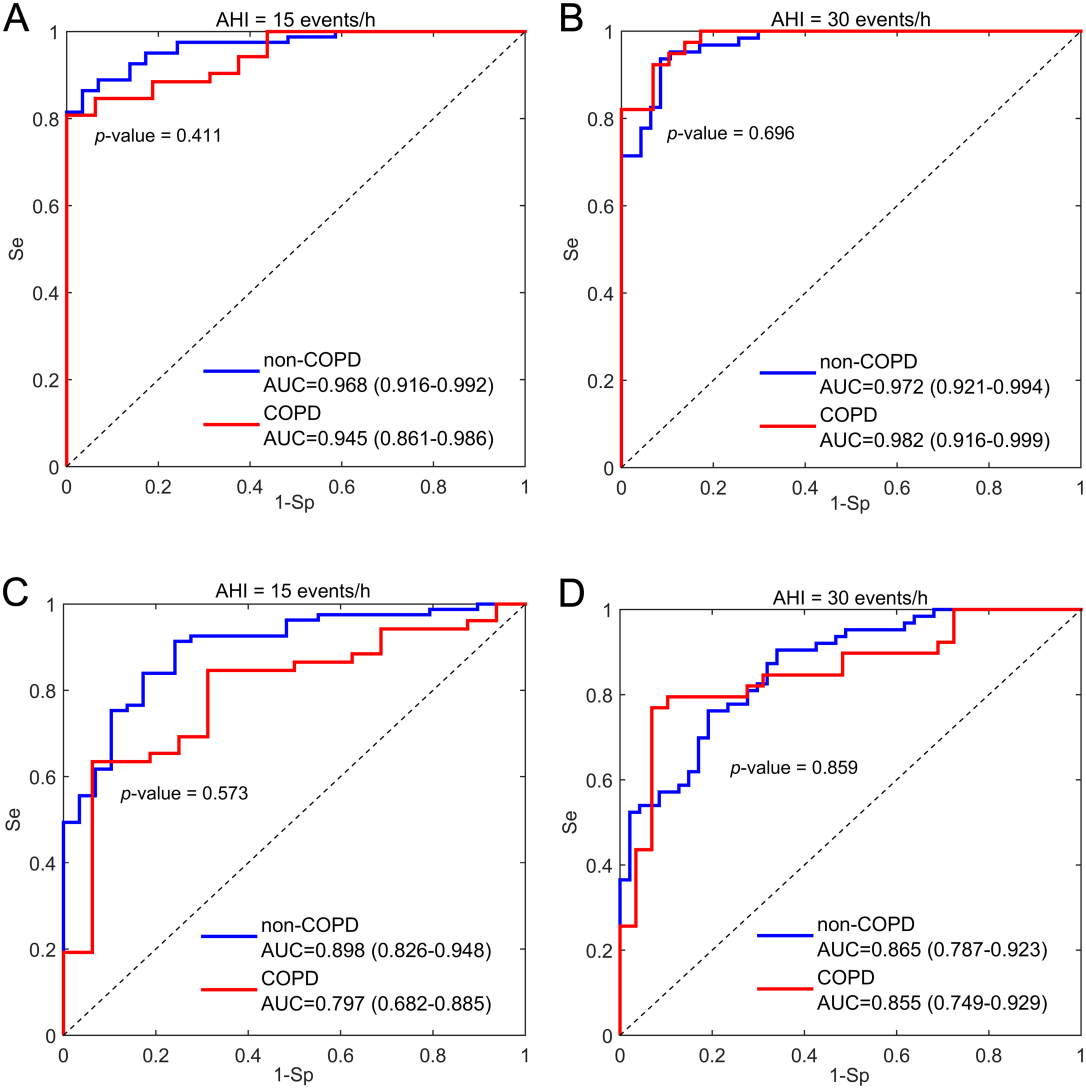
Receiver operating characteristics curves of the estimated AHI. (A) Supervised portable oximetry in the hospital using a cutoff of AHI ≥15 events/h. (B) Supervised portable oximetry in the hospital using a cutoff of AHI ≥30 events/h. (C) Unattended portable oximetry at home using a cutoff of AHI ≥15 events/h. (D) Unattended portable oximetry at home using a cutoff of AHI ≥30 events/h. AHI: apnea-hypopnea index from standard PSG; non-COPD: patients without chronic obstructive pulmonary disease; COPD: patients with chronic obstructive pulmonary disease; AUC: area under the ROC curve.

Regarding differences between non-COPD and COPD patients in terms of the effectiveness of the simplified oximetry-based test for OSAS, Fig 5 shows the ROC curves for both groups in the hospital and in the unsupervised setting at home. Notice that no significant differences were found between the curves of both groups for the cutoffs under study.

Tables 5 and 6 summarize the performance assessment of the MLP_OX-LAB_ and conventional ODIs for the groups under study in the hospital setting. For each individual cutoff, the estimated AHI_OX-LAB_ using the proposed MLP ANN reached high similar accuracy both in the non-COPD and the COPD groups (87.3% *vs*. 86.8%, and 92.7% *vs*. 91.2% for 15 and 30 events/h, respectively). On the other hand, conventional ODIs showed notably higher variability between non-COPD and COPD groups.

**Table 5:** Diagnostic performance of the proposed MLP_OX-LAB_ and conventional ODIs from in-laboratory portable oximetry (simultaneously to the PSG) in the non-COPD test group.

**Moderate OSAS (AHI ≥15 events/h)**							
	**Se (%)**	**Sp (%)**	**PPV (%)**	**NPV (%)**	**LR+**	**LR-**	**Acc (%)**
AHI_OX-LAB_	97.5	58.6	86.8	89.5	2.36	0.04	87.3
	(91.4–99.7)	(38.9–76.5)	(78.1–93.0)	(66.9–98.7)	(1.5–3.6)	(0.01–0.2)	(80.0–92.7)
ODI3	91.4	100	100	80.6	*NA*^a^	0.09	93.6
	(83.2–99.0)	(-)	(-)	(63.4–97.5)	(-)	(0.01–0.17)	(87.6–99.3)
ODI4	69.1	100	100	53.7	*NA*^a^	0.31	77.3
	(56.6–81.5)	(-)	(-)	(38.0–69.7)	(-)	(0.19–0.43)	(67.7–86.5)
**Severe OSAS (AHI ≥30 events/h)**							
	**Se (%)**	**Sp (%)**	**PPV (%)**	**NPV (%)**	**LR+**	**LR-**	**Acc (%)**
AHI_OX-LAB_	95.2	89.4	92.3	93.3	8.95	0.05	92.7
	(86.7–99.0)	(76.9–96.5)	(83.0–97.5)	(81.7–98.6)	(3.9–20.5)	(0.02–0.2)	(86.4–96.4)
ODI3	76.2	100	100	75.8	*NA*^a^	0.24	86.4
	(62.5–88.7)	(-)	(-)	(61.7–87.8)	(-)	(0.11–0.38)	(78.2–93.9)
ODI4	41.3	100	100	56.0	*NA*^a^	0.59	66.4
	(26.4–56.6)	(-)	(-)	(42.8–68.4)	(-)	(0.43–0.74)	(55.2–76.7)

Data are presented as magnitude and 95% confidence interval. AHI: apnea-hypopnea index; Se: sensitivity; Sp: specificity; PPV: positive predictive value; NPV: negative predictive value; LR+: positive likelihood ratio; LR-: negative likelihood ratio; Acc: accuracy; AHI_OX-LAB_: estimated AHI from in-laboratory nocturnal oximetry; ODI3: oxygen desaturation index of 3%; ODI4: oxygen desaturation index of 4%.

^a^ *NA*: if Sp = 100%, then LR+ is not defined.

**Table 6:** Diagnostic performance of the proposed MLP_OX-LAB_ and conventional ODIs from in-laboratory portable oximetry (simultaneously to the PSG) in the COPD test group.

**Moderate OSAS (AHI ≥15 events/h)**							
	**Se (%)**	**Sp (%)**	**PPV (%)**	**NPV (%)**	**LR+**	**LR-**	**Acc (%)**
AHI_OX-LAB_	96.2	56.3	87.7	81.8	2.20	0.07	86.8
	(86.8–99.5)	(29.9–80.2)	(76.3–94.9)	(48.2–97.7)	(1.3–3.8)	(0.02–0.3)	(76.5–94.1)
ODI3	88.5	87.5	95.8	70.0	7.08	0.13	88.2
	(77.5–98.6)	(66.7–100)	(88.2–100)	(43.7–96.3)	(2.42–12.13)	(0.02–0.27)	(79.1–96.1)
ODI4	73.1	100	100	53.3	*NA*^a^	0.27	79.4
	(58.8–87.2)	(-)	(-)	(30.5–75.3)	(-)	(0.13–0.41)	(68.1–90.4)
**Severe OSAS (AHI ≥30 events/h)**							
	**Se (%)**	**Sp (%)**	**PPV (%)**	**NPV (%)**	**LR+**	**LR-**	**Acc (%)**
AHI_OX-LAB_	97.4	82.8	88.4	96.0	5.65	0.03	91.2
	(86.5–99.9)	(64.2–94.2)	(74.9–96.1)	(79.6–99.9)	(2.5–12.6)	(0.004–0.2)	(80.9–95.6)
ODI3	92.3	93.1	94.7	90.0	13.39	0.08	92.7
	(81.2–100)	(80.8–100)	(85.2–100)	(75.6–100)	(4.50–20.30)	(0–0.20)	(84.6–99.5)
ODI4	53.9	100	100	61.7	*NA*^a^	0.46	73.5
	(33.5–74.0)	(-)	(-)	(43.4–79.5)	(-)	(0.26–0.67)	(59.2–86.5)

Data are presented as magnitude and 95% confidence interval. AHI: apnea-hypopnea index; Se: sensitivity; Sp: specificity; PPV: positive predictive value; NPV: negative predictive value; LR+: positive likelihood ratio; LR-: negative likelihood ratio; Acc: accuracy; AHI_OX-LAB_: estimated AHI from in-laboratory nocturnal oximetry; ODI3: oxygen desaturation index of 3%; ODI4: oxygen desaturation index of 4%.

^a^ *NA*: if Sp = 100%, then LR+ is not defined.

Tables 7 and 8 summarize the diagnostic assessment of the MLP_OX-HOME_ and conventional ODIs in the unattended setting at home. Regarding the estimated AHI using the proposed MLP ANN, moderate accuracies were achieved in both populations: 78.2% *vs*. 75.0%_,_ and 76.4% *vs*. 77.9% in non-COPD and COPD groups for cutoffs of 15 and 30 events/h, respectively. Imbalance in the sensitivity–specificity pair increased compared to the hospital setting thought remained similar in both patient groups. On the other hand, conventional ODI3 and ODI4 showed the common higher specificity inherent to oximetry as well as a notably higher variability between patients with and without COPD.

**Table 7:** Diagnostic performance of the proposed MLP_OX-HOME_ and conventional ODIs from portable oximetry at home (unattended in a different preceding/consecutive night to PSG) in the non-COPD test group.

**Moderate OSAS (AHI ≥15 events/h)**							
	**Se (%)**	**Sp (%)**	**PPV (%)**	**NPV (%)**	**LR+**	**LR-**	**Acc (%)**
AHI_OX-HOME_	97.5	24.1	78.2	77.8	1.29	0.10	78.2
	(91.4–99.7)	(10.3–43.5)	(68.9–85.8)	(40.0–97.2)	(1.0–1.6)	(0.02–0.5)	(69.1–84.6)
ODI3	59.3	93.1	96.0	45.0	8.59	0.44	68.2
	(46.4–71.9)	(80.7–100)	(88.3–100)	(30.2–60.1)	(2.81–13.66)	(0.30–0.60)	(57.6–78.3)
ODI4	45.7	100	100	39.7	*NA*^a^	0.54	60.0
	(32.7–58.6)	(-)	(-)	(26.7–52.3)	(-)	(0.41–0.67)	(49.2–70.4)
**Severe OSAS (AHI ≥30 events/h)**							
	**Se (%)**	**Sp (%)**	**PPV (%)**	**NPV (%)**	**LR+**	**LR-**	**Acc (%)**
AHI_OX-HOME_	81.0	70.2	78.5	73.3	2.72	0.27	76.4
	(69.1–89.8)	(55.1–82.7)	(66.5–87.7)	(58.1–85.4)	(1.7–4.3)	(0.2–0.5)	(67.3–83.6)
ODI3	44.4	97.9	96.6	56.8	20.89	0.57	67.3
	(29.3–59.0)	(92.9–100)	(86.9–100)	(43.5–69.3)	(4.84–18.42)	(0.42–0.73)	(56.3–77.1)
ODI4	23.8	97.9	93.8	48.9	11.19	0.78	55.5
	(12.0–36.2)	(92.9–100)	(76.8–100)	(36.6–61.2)	(2.12–11.99)	(0.65–0.91)	(44.4–66.8)

Data are presented as magnitude and 95% confidence interval. AHI: apnea-hypopnea index; Se: sensitivity; Sp: specificity; PPV: positive predictive value; NPV: negative predictive value; LR+: positive likelihood ratio; LR-: negative likelihood ratio; Acc: accuracy; AHI_OX-HOME_: estimated AHI from at-home nocturnal oximetry; ODI3: oxygen desaturation index of 3%; ODI4: oxygen desaturation index of 4%.

^a^ *NA*: if Sp = 100%, then LR+ is not defined.

**Table 8:** Diagnostic performance of the proposed MLP_OX-HOME_ and conventional ODIs from portable oximetry at home (unattended in a different preceding/consecutive night to PSG) in the COPD test group.

**Moderate OSAS (AHI ≥15 events/h)**							
	**Se (%)**	**Sp (%)**	**PPV (%)**	**NPV (%)**	**LR+**	**LR-**	**Acc (%)**
AHI_OX-HOME_	86.5	37.5	81.8	46.2	1.39	0.36	75.0
	(74.2–94.4)	(15.2–64.6)	(69.1–90.9)	(19.2–74.9)	(0.9–2.1)	(0.1–0.9)	(63.2–83.8)
ODI3	69.2	93.8	97.3	48.4	11.08	0.33	75.0
	(54.2–84.5)	(76.8–100)	(90.4–100)	(26.5–70.5)	(2.44–11.36)	(0.16–0.52)	(62.7–87.2)
ODI4	48.1	93.8	96.2	35.7	7.69	0.55	58.8
	(31.3–65.9)	(76.8–100)	(85.6–100)	(18.2–54.2)	(1.68–7.80)	(0.36–0.77)	(44.8–73.4)
**Severe OSAS (AHI ≥30 events/h)**							
	**Se (%)**	**Sp (%)**	**PPV (%)**	**NPV (%)**	**LR+**	**LR-**	**Acc (%)**
AHI_OX-HOME_	84.6	69.0	78.6	76.9	2.73	0.22	77.9
	(69.5–94.1)	(49.2–84.7)	(63.2–89.7)	(56.4–91.0)	(1.6–4.8)	(0.1–0.5)	(67.0–86.8)
ODI3	61.5	96.6	96.0	65.1	17.85	0.40	76.5
	(42.0–80.0)	(88.0–100)	(84.9–100)	(47.2–82.8)	(4.15–18.10)	(0.21–0.61)	(63.9–87.8)
ODI4	35.9	96.6	93.3	52.8	10.41	0.66	61.8
	(17.5–55.0)	(88.0–100)	(76.3–100)	(36.6–69.2)	(1.99–10.50)	(0.46–0.87)	(47.4–75.9)

Data are presented as magnitude and 95% confidence interval. AHI: apnea-hypopnea index; Se: sensitivity; Sp: specificity; PPV: positive predictive value; NPV: negative predictive value; LR+: positive likelihood ratio; LR-: negative likelihood ratio; Acc: accuracy; AHI_OX-HOME_: estimated AHI from at-home nocturnal oximetry; ODI3: oxygen desaturation index of 3%; ODI4: oxygen desaturation index of 4%.

## Discussion

In the present study, portable oximetry was assessed as a simplified tool for OSAS diagnosis in COPD patients. In order to carry out a thorough analysis, portable oximetry was tested both in the hospital and at home. In addition, two independent populations were assessed: (i) non-COPD subjects and (ii) COPD patients. Our results revealed that no significant differences exist between COPD and non-COPD patients concerning the capability of the proposed ANN from portable oximetry as a screening test for OSAS.

In our aim to maximize the diagnostic ability of portable oximetry, we proposed the automated analysis of oximetric recordings by means of a regression MLP ANN. In this regard, ANNs have been widely applied in medical applications to expedite decisions and avoid misdiagnosis. Particularly, MLP is probably the most popular ANN and it has demonstrated to be very useful in the framework of OSAS management [23, 24, 26, 47, 48]. The study by Marcos et al. [26] supports the usefulness of a regression-based MLP ANN for estimating the AHI using supervised SpO_2_ recordings from in-hospital PSG. In the present study, we assessed the accurateness and reliability of portable oximetry and ANNs to screen for OSAS at COPD patients’ home.

In the context of OSAS detection from oximetry, simpler oximetric indices, such as the average number of desaturations (conventional ODIs), have been proposed [31, 49, 50]. Nevertheless, a systematic underestimation of the disease has been reported and the performance notably varies among studies [4, 51, 52]. In the present research, we proposed a complex approach based on ANNs. Our experiments found that the estimated AHI from the MLP ANN significantly outperformed conventional ODIs in terms of agreement with actual AHI from PSG both in the hospital and at home. Furthermore, the ANN reached similar diagnostic performance in non-COPD and COPD groups in both settings (87.3% *vs*. 86.8% Acc for AHI ≥15 events/h and 92.7% *vs*. 91.2% Acc for AHI ≥30 events/h in the hospital; 78.2% *vs*. 75.0% Acc for AHI ≥15 events/h and 76.4% *vs*. 77.9% Acc for AHI ≥30 events/h at home), notably more consistent than conventional ODI3 (93.6% *vs*. 88.2% Acc for AHI ≥15 events/h and 86.4% *vs*. 92.7% Acc for AHI ≥30 events/h in the hospital; 68.2% *vs*. 75.0% Acc for AHI ≥15 events/h and 67.3% *vs*. 76.5% Acc for AHI ≥30 events/h at home) and ODI4 (77.3% *vs*. 79.4% Acc for AHI ≥15 events/h and 66.4% *vs*. 73.5% Acc for AHI ≥30 events/h in the hospital; 60.0% *vs*. 58.8% Acc for AHI ≥15 events/h and 55.5% *vs*. 61.8% Acc for AHI ≥30 events/h at home). In addition, once optimized and trained, ANNs are reliable, easy-to-use, and computationally efficient tools, which are major features in order to speed up decision-making.

Pertinent features derived from each overnight oximetric profile fed the neural network. Previous studies showed that these variables have high discriminant ability between OSAS-negative and OSAS-positive patients in the context of binary classification from supervised oximetry [32–34]. In the present study, we assessed the capability of these features to predict the AHI from portable oximetry. The proposed FCBF technique for variable selection automatically identified the features most representative of the actual AHI from PSG (higher symmetrical uncertainty) as well as the less redundant (lower symmetrical uncertainty between each pair of variables). It is remarkable that *M1t*, *M3t*, *M4t*, *P*_*R*_, *SampEn*, *CTM*, and *LZC* were all included in the optimum feature subset both in the hospital and at home, which evidences the relevancy of these features in the regression-based ANN for AHI estimation. They gather complementary information from the oximetry signal (time: *M1t*, *M3t*, *M4t*; frequency: *P*_*R*_; and nonlinear: *SampEn*, *CTM*, and *LZC*) in order to account for all the changes linked with apneic events which are commonly quantified by means of the AHI.

In regard to the agreement between estimated and actual AHI, a small similar overestimation can be seen for the groups under study both in the hospital (1.30 non-COPD *vs*. 1.60 COPD) and at home (0.61 non-COPD *vs*. 0.73 COPD). Nevertheless, the diagnostic performance did not agree with this slight overall overestimation, as can be derived from Tables 5–8. In both settings, higher sensitivity than specificity was obtained for the groups under study, especially for the lower cutoff (AHI ≥15 events/h), which suggests a systemic misclassification into a higher severity group. The Bland-Altman plots in Fig 3A and 3B show different trends for low and high AHI values whatever the group or the setting under analysis. Significant overestimation was obtained for AHI under 15 events/h, which explains the misclassification towards higher severity classes. Conversely, an important underestimation is observed for higher AHI values, which compensates for the initial trend leading to a slight average overestimation. It is important to note that this behavior can be observed both in the hospital and at home regardless of suffering from COPD, which agrees with our initial hypothesis.

In our aim to gain insight into the usefulness of at-home portable oximetry for OSAS screening in COPD, a thoroughly analysis of misclassified COPD patients in the unsupervised setting was accomplished. Traditionally, it has been suggested that profound nocturnal desaturations characteristic of COPD could lead to increased misdiagnosis in oximetry-based screening tests for OSAS. Nevertheless, our findings revealed no influence on the diagnostic capability of oximetry linked with transient worsening due to COPD. Furthermore, no differences related to the severity of the pulmonary disease were found among misclassified patients within the COPD group at home. Regarding mild COPD (GOLD 1), four patients showing mild OSAS were misclassified as severe using AHI_OX-HOME_. Two patients showed BMI >29 kg/m^2^ and OSAS was also overestimated using in-laboratory portable oximetry. Analyzing the remaining two patients, both were hypertensive and one had a BMI of 50.5 kg/m^2^. In regard to moderate COPD patients (GOLD 2), just one non-OSAS subject (borderline with AHI_PSG_ = 4.2 events/h, obese, and hypertensive) was misclassified as mild OSAS both at home and in the hospital. In addition, six OSAS patients were misclassified at home. It is important to note that all cases showed significantly decreased (5 patients) or increased (1 patient) number of desaturations in the unattended setting compared to in-hospital recordings, which could be due to the well-known night-to-night variability of OSAS. Regarding severe COPD (GOLD 3), four OSAS patients were incorrectly classified into a category of higher severity (3 from mild to moderate and 1 from moderate to severe). Two mild OSAS patients showed poor sleep efficiency in the hospital (<55%) and little time in REM sleep, which could hide the actual severity of the disease. The moderate OSAS patient showed overweight (BMI = 29.4 kg/m^2^). On the other hand, two severe COPD subjects were wrongly classified at home into an OSAS category of lower severity. Both patients showed significant contribution of positional apneas during PSG and they presented markedly low number of desaturations at home due to night-to-night variability.

During the last years, researchers have made a great effort on the development of efficient screening tools for OSAS based on portable monitoring in order to expedite diagnosis. Nevertheless, the validation of almost all simplified screening tests excluded patients with significant comorbidities, such as cardiovascular or pulmonary diseases. Therefore, data on the accuracy of abbreviated methods in COPD patients are very limited [13, 16, 17]. In fact, the AASM does not recommend its use on such populations due to the lack of appropriate evidences supporting their accuracy [11]. Table 9 shows the results reported in the scarce studies where portable monitors are assessed for OSAS screening in COPD patients. In the work by Pépin et al. [20], the delta index was proposed to characterize OSAS from portable oximetric recordings acquired in the hospital. The authors obtained promising results though a small population composed of only 8 COPD patients was analyzed.

**Table 9:** Evidences on the effectiveness of portable oximetry monitoring for OSAS detection in patients with COPD in the state-on-the-art and in the present study.

Author	Population	Method and setting	Goal	Se (%)	Sp (%)	ICC
Pépin *et al*. (1991) [20]	26 patients 15 OSAS 8 COPD 3 Restrictive	Method: Delta index Setting: In-hospital portable oximetry	OSAS detection in: Whole population Patients showing basal SpO_2_ <93% COPD patients	75 100 100	86 83 100	- - -
Oliveira *et al*. (2012) [21]	26 COPD patients showing symptoms of suffering from OSAS	Method: Manual AHI Setting. SpO_2_ from RP in-lab and at home	Agreement: PSG *vs*. RP_LAB_ PSG *vs*. RP_HOME_ RP_LAB_ *vs*. RP_HOME_	- - -	- - -	0.61 0.47 0.47
Scott *et al*. (2014) [22]	59 COPD (GOLD 3–4)	Method: Visual and automated analyses Setting: In-hospital portable oximetry	OSAS detection (AHI ≥15) Visual inspection Automated ODI4	59 60	60 63	- -
Present study (2016)	371 patients showing symptoms of suffering from OSAS 193 training 178 test (110 non-COPD and 68 COPD)	Method: Automated analyses by MLP ANN Setting: Portable oximetry in-lab and at home	OSAS detection in the hospital (AHI ≥15): non-COPD COPD OSAS detection at home (AHI ≥15): non-COPD COPD Agreement PSG *versus* MLP_OX-LAB_ in the hospital non-COPD COPD Agreement PSG *versus* MLP_OX-HOME_ at home non-COPD COPD	97.5 96.2 97.5 86.5 - - - -	58.6 56.3 24.1 37.5 - - - -	- - - - 0.94 0.94 0.73 0.79

OSAS: obstructive sleep apnea syndrome; COPD: chronic obstructive pulmonary disease; GOLD: Global Initiative for Chronic Obstructive Lung Disease; AHI: apnea-hypopnea index; SpO_2_: blood oxygen saturation; RP: respiratory polygraphy; PSG: polysomnography; RP_LAB_: in-hospital respiratory polygraphy; RP_HOME_: respiratory polygraphy at home; ODI4: oxygen desaturation index of 4%; MLP_OX-LAB_: multilayer perceptron artificial neural network trained with oximetric recordings from portable oximetry in the hospital; MLP_OX-HOME_: multilayer perceptron artificial neural network trained with oximetric recordings from portable oximetry at home.

In the study by Oliveira et al. [21], a Type 3 portable monitor was used, which was assessed in a population composed of 26 COPD patients. The authors reported a moderate ICC equal to 0.61 between the actual AHI from PSG and manual scoring of in-hospital portable recordings, whereas the agreement decreases up to 0.47 when the monitoring were carried out at home.

In a recent study by Scott et al. [22] a portable oximeter was tested in the hospital to determine the presence or absence of OSAS in 59 COPD patients. Using a cutoff of 15 events/h for positive OSAS, visual inspection of the overnight oximetric profile reached 59% Se and 60% Sp, while the performance increased up to 60% Se and 63% Sp using the automated ODI4.

In the present research, additional knowledge is provided on the usefulness of automated processing of portable oximetry as a single tool for screening for OSAS in COPD patients. One of the main novelties of our study is that the impact of suffering from COPD on the capability of portable oximetry was assessed by the analysis of two populations: non-COPD and COPD independent test datasets. In addition, the proposed methodology based on automated pattern recognition using ANNs was validated both in the hospital and at home. Regarding portable monitoring in a supervised setting, our automated approach improved the performance reached by Scott et al. (60% Se– 63% Sp vs. 96.2% Se– 56.3% Sp, AHI ≥15 events/h) [22]. On the other hand, our sensitivity–specificity pair showed higher imbalance. In the unattended setting, the agreement with the AHI from PSG reported by Oliveira et al. [21] was also notably enhanced (0.47 ICC vs. 0.79 ICC). Essential differences between the present research and previous studies account for the dissimilarities in the diagnostic capability of oximetry. Firstly, datasets of different size and severity were studied. In this regard, it is important to highlight that our sample size is larger than populations analyzed in previous studies and accounts for a wide range of severities in both OSAS and COPD. Secondly, there are major methodological differences among studies. Previous researchers characterized the portable recording by means of conventional indices, such as the delta index [20] or ODI4 [22], or proposed visual inspection of the overnight oximetric profile [22] or manual scoring of respiratory events [21]. On the other hand, our proposal is based on advanced signal processing techniques aimed at deriving as much information as possible from oximetry in order to maximize its diagnostic ability. In addition, the most relevant and complementary features were identified and high-performance pattern recognition techniques were used to optimally manage all these data.

Some limitations of the study should be discussed. We analyzed a large population of patients showing clinical suspicion of OSAS. Particularly, the group composed of patients with concomitant COPD was larger than in previous similar studies aimed at assessing portable oximetry in the presence of comorbidities found in the state-of-the-art. Nevertheless, the number of patients with very severe COPD (GOLD 4) could be larger in order to generalize our results. Regarding the composition of the population under study, the great imbalance between the number of non-OSAS and OSAS patients is an additional drawback that merits some discussion. Sleep apnea patients were predominant, particularly severe OSAS (56.0% of patients in the training set, 57.3% in the non-COPD group, and 57.4% in the COPD group). This agrees with the prevalence of the disease reported in similar studies [53]. However, this imbalance could influence our results. Although we used a regression-based approach and small bias was obtained in the estimation of the AHI from portable oximetry, unbalanced sensitivity-specificity pairs (sensitivity notably higher than specificity) were obtained when using a fixed diagnostic threshold, particularly for the cutoff AHI ≥15 events/h, where the imbalance is more significant. While the proposed ANNs could be optimized in future studies using a more balanced population, our models provide interesting insight into the usefulness of portable monitoring for OSAS detection in COPD patients, i.e., oximetry could be an efficient tool as a simplified screening test for OSAS regardless of suffering from COPD.

An additional limitation is linked with conducting the unsupervised oximetry at home and the reference PSG in different nights. The night-to-night variability of OSAS is widely known, which could indirectly increase the differences between the actual AHI from PSG and the AHI estimated using portable oximetry at home in a different night. Clearly, the agreement between studies conducted simultaneously (PSG and in-lab oximetry) is expected to be higher than the agreement between measures derived from studies performed separately (PSG and at-home oximetry). Therefore, the decrease in the agreement between the actual AHI and the estimated AHI from unattended oximetry in the home setting, as well as the decrease in the diagnostic performance, could be mostly due to this issue. Nonetheless, both the non-COPD and the COPD groups showed similar behavior and no significant differences were found between groups, which confirm our initial hypothesis.

## Conclusions

A simplified screening test for OSAS based on automated analysis of portable oximetry by means of a regression ANN exhibited no significant differences between non-COPD and COPD patients in terms of diagnostic performance. The diagnostic accuracy of the estimated AHI from oximetry and its agreement with the actual AHI from PSG were similar regardless of the presence of COPD both in the laboratory and at home. Furthermore, in the home setting, the proposed regression ANN performed similar in patients with such comorbid condition regardless of the severity of COPD, particularly in stages GOLD 1 to 3. Our results suggest that automated analysis of unsupervised portable oximetry at home may be recommended as an efficient tool for moderate-to-severe OSAS diagnosis also in the presence of COPD.

## Supporting information

S1 Table Actual AHI from PSG, ODI3, ODI4, and estimated AHI from the proposed oximetry-based MLP ANN both in the hospital and at home of each subject in the non-COPD test dataset. (XLSX)

S2 Table Actual AHI from PSG, ODI3, ODI4, and estimated AHI from the proposed oximetry-based MLP ANN both in the hospital and at home of each subject in the COPD test dataset. (XLSX)
